# Wanted

**Published:** 1884-12

**Authors:** 


### Wanted.

Three or four copies of the Transactions of the American Dental Association, for the year 1870. Also one copy each of the following years: 1859-60-62-63, and 1876. Any one having these copies, and willing to part with them, will please send them to the Editor of the DENTAL REGISTER, for which ample compensation will be made. Also, Transactions of the Illinois State Dental Society, for the years 1873–4, and 1876–9. Any one having these volumes and willing to part with them, will also please send them. We shall hope to receive these at an early day.

